# Sweet syndrome: a rare feature of ANCA-associated vasculitis or unusual consequence of azathioprine-induced treatment

**DOI:** 10.1186/s13223-018-0265-6

**Published:** 2018-11-08

**Authors:** A. U. Arun Kumar, Mohamed E. Elsayed, Ahmed Alghali, Alaa A. Ali, Husham Mohamed, Wael Hussein, Catriona Hackett, Niamh Leonard, Austin G. Stack

**Affiliations:** 1 Division of Nephrology, Department of Medicine, University Hospital Limerick, St Nessans Rd, Limerick, Ireland; 2 Graduate Entry Medical School, University of Limerick, Limerick, Ireland; 3 Division of Dermatology, Department of Medicine, University Hospital Limerick, Limerick, Ireland; 4 Department of Pathology, St James Hospital, Dublin, Ireland; 5 Health Research Institute, University of Limerick, Limerick, Ireland

## Abstract

**Background:**

Sweet syndrome is a rare skin condition characterised by fever, neutrophilia, and tender erythematous skin lesions and has been reported to occur in association with anti-neutrophil cytoplasmic antibodies (ANCA) as well as complicate treatment with azathioprine therapy. Azathioprine, a relatively safe immunosuppressive, is frequently used to maintain disease remission in the treatment of ANCA-associated vasculitis. The occurrence of Sweet syndrome in a patient with ANCA-positive vasculitis and following treatment with azathioprine prompted us to present this clinical case and share this unusually rare occurrence. In doing so, we also wish to discuss current understanding of the disease and plausible associations.

**Case presentation:**

Herein, we discuss the case of a 54-year old white male, who presented with features of ANCA vasculitis with haemoptysis, arthralgia, abnormal kidney function with active urine sediment and a positive p-ANCA titre. Standard immunosuppressive treatment with corticosteroids and intravenous rituximab resulted in disease remission. Due to significant steroid side effects, his steroid treatment was gradually tapered and switched to azathioprine over a 6-month period. Two weeks following initiation of azathioprine, he developed a painful maculo-papular erythematous skin rash and fever. A skin biopsy confirmed classical features consistent with Sweet syndrome. Withdrawal of azathioprine and treatment with oral corticosteroids and colchicine therapy resulted in complete resolution of the rash, although he continued to have high titres of MPO positive ANCA.

**Conclusion:**

Sweet syndrome is a rare adverse reaction to azathioprine but has also been reported to occur in association with ANCA vasculitis. The temporal association with azathioprine in our case and the relatively rapid resolution of the skin vasculitis upon its withdrawal suggested a primarily drug-induced reaction rather than an associated feature of ANCA vasculitis.

## Background

Anti-neutrophil cytoplasmic antibody-associated vasculitis (ANCA-V) is a group of diseases characterised by inflammation and necrosis of small and medium sized-blood vessels [1]. Azathioprine (AZA) is commonly used in the maintenance phase of this potentially life threatening disease in order to maintain remission after induction therapy [2]. Sweet syndrome, an acute febrile neutrophilic dermatosis, is a rare hypersensitivity reaction that has been reported to occur following exposure to azathioprine and AZA induced Sweet syndrome was first described in 1995 [3]. To-date, a total of 18 cases have been reported after that first description [4]. In the majority of these cases, exposure to AZA treatment occurred in the setting of inflammatory bowel disease. Sweet syndrome has also been reported in association with ANCA-V as highlighted in a recent French multi-centre study [5]. To our knowledge, there are only two published case reports of AZA-induced Sweet syndrome in the literature where the underlying condition was ANCA-V [6, 7]. Causality is a difficult construct to prove in the circumstances of single isolated clinical cases. However, clearly defining the temporal sequence of events between the exposure and a specific outcome is suggestive of a strong association, especially if the condition disappears when the offending culprit is removed.

## Case report

A 53-year old white male was referred to University Hospital Limerick with a macular rash on extensor aspects of upper limb and torso, bilateral loin pain, arthralgia, fatigue, active urinary sediment and acute kidney injury in August 2015. The current presentation was preceded by two previous episodes of illness in which he had reported similar symptoms along with haemoptysis in April and July 2014. Past medical history revealed the presence of a peripapilary melanoma of the left eye treated with radiotherapy in 2010 and a basal cell carcinoma of the mid-back excised in 2000. The patient denied tobacco use and drank occasionally and denied any family history off kidney disease. He worked on a farm and was married with two children. On presentation his blood pressure was 124/70 mmHg, weight 91 kg, and there was evidence of macular rash on his back but no lower limb oedema. Urine evaluation demonstrated activity with 3+ protein and 3+ blood, and his serum creatinine was elevated at 128 μmol/L compared to a baseline of 116 μmol/L recorded in April 2014. Serology was positive for P-ANCA with a titre of 160 and he had an anti-MPO titre of over 200 units/mL; apart from this ANA was positive with a titre of 1600 with negative Anti-dsDNA, Anti-Sm, Anti-Sm/RNP and Anti-SSB/RO/LA; Serology for HIV 1 + 2 Ag/Ab and Hepatitis BsAg & Hepatitis C antibody were negative; complement levels were within normal range C3 of 0.82 g/L and C4 of 0.24 g/L. ESR was 30 mm/h and Hs-CRP was 48 mg/L; rest of his routine bloods were unremarkable (White cell count 5.8 × 10^9^/L, haemoglobin 13.5 g/dL, neutrophils 4.00 × 10^9^/L, platelet 210 × 10^9^/L, sodium 141 mmol/L, potassium 4.2 mmol/L, total protein 69 g/L, albumin 39 g/L, serum calcium 2.31 mmol/L, serum phosphate 1.09 mmol/L, bilirubin 7.3 µmol/L, alkaline phosphatase 81 IU/L, gamma-glutamyl transferase 25 IU/L, alanine-aminotransferase 39 IU/L, prothrombin time 12.0 s, activated partial thromboplastin time 32.0 s). Chest X-ray was normal. A recent ultrasound of his kidneys demonstrated normal size and shape with normal cortex. A native kidney biopsy revealed evidence of moderate arteriosclerosis with 30–35% fibrosis, areas of thrombotic microangiopathy but without evidence of fibrinoid necrosis or epithelial crescents. Immunofluorescence was negative and electron microscopy revealed thin glomerular basement membranes with mean measurements less than 250 nM and many measurements less than 200 nM, consistent with thin basement membrane disease (TBMD).

Based on the presence of an AKI with active urine sediment, associated skin rash, and positive serology with high titres of P-ANCA and anti-MPO along with an antecedent history of haemoptysis, we diagnosed an ANCA-associated vasculitis likely microscopic polyangiitis (MPA) based on the European Medicines Agency Algorithm, although the kidney biopsy sample failed to demonstrate classical features of renal vasculitis [8]. Treatment was initiated with corticosteroids and intravenous rituximab infusions (at day 0 and day 18) with prophylaxis for pneumocystis (co-trimoxazole), osteoporosis (oral Vitamin D3 and bisphosphonates), and gastritis (proton pump inhibitor). Four weeks later his urine sediment normalised with no detectable blood or protein; his CRP fell to < 5 mg/L and his ANCA titres fell to 40 but his anti-MPO level remained remarkably high 198 IU/mL. Two months later, he developed severe depression attributed to steroids requiring taper and the initiation of anti-psychotic therapy.

**Fig. 1:**
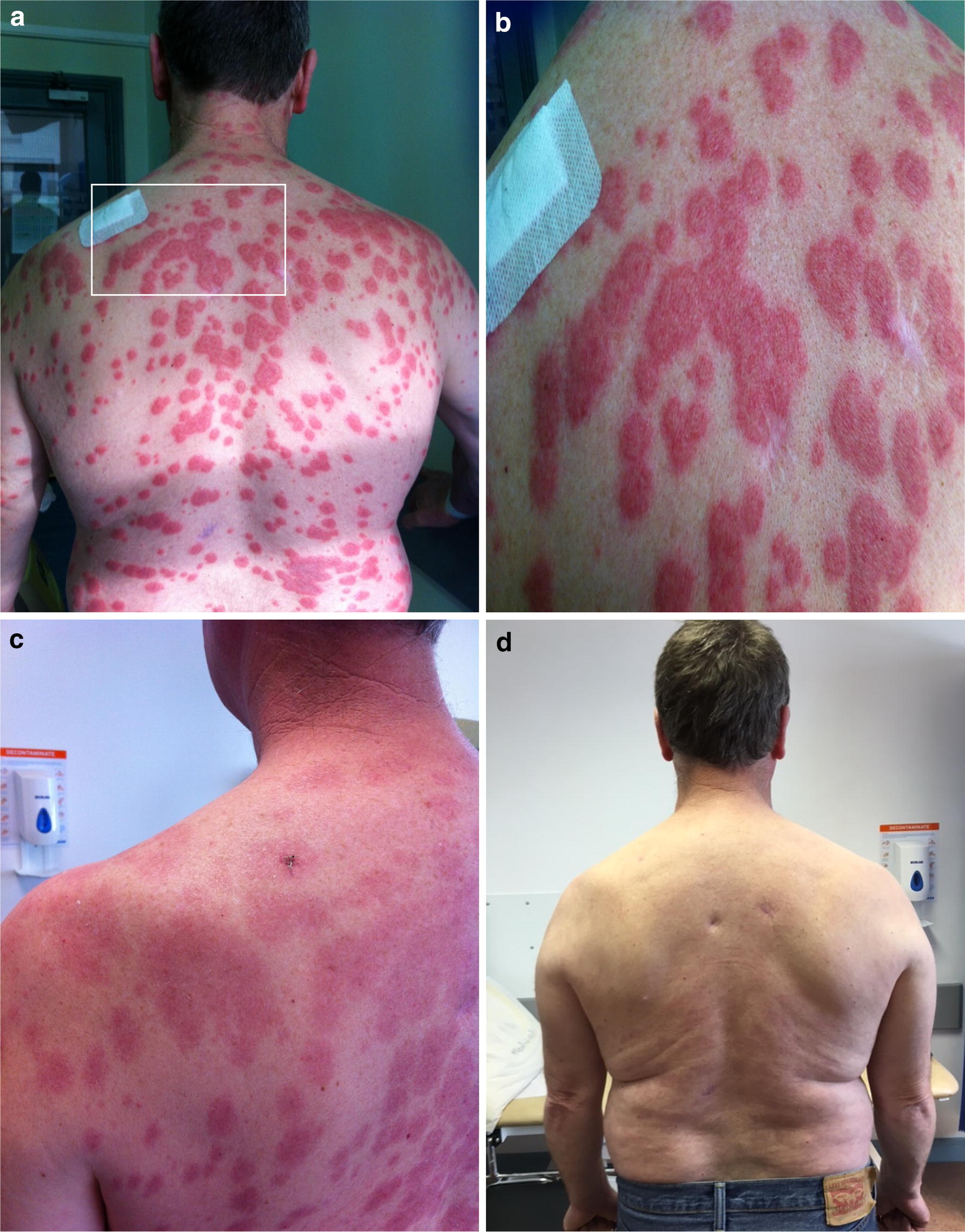
**a** Abrupt onset of disseminated painful skin lesions. **b** Target lesions visible with areas of confluence. **c** After 2 weeks of steroid therapy and AZA withdrawal. **d** After 4 weeks of steroids and addition of colchicine

Six months following initial presentation, his vasculitis remained clinically quiescent and the patient was commenced on azathioprine (AZA) as a steroid sparing agent for maintenance immunosuppression. Two weeks after AZA commencement, the patient presented to the emergency department with acute painful disseminated morbilliform rash along with fever and myalgia. On examination temp was 39.7 °C and a widespread indurated erythematous papular rash was noted (Fig. 1a, b). An extensive viral and immunologic work up was negative including virology for herpes zoster, and simplex. Considering his history of immunosuppression he was empirically started on acyclovir. Full blood count revealed neutrophilic leucocytosis with neutrophil count of 12.2 × 10^9^/L, Hs-CRP 259 and ESR 59, and a stable serum creatinine concentration of 115 μmol/L. A full septic work up was negative including negative serology for HIV, hepatitis A, B, C, and CMV. A skin biopsy was diagnostic of Sweet’s syndrome, in which the affected lesions revealed extensive neutrophilic infiltrate in the dermis but without evidence of leukocytoclastic vasculitis, herpes, zoster, or erythema multiform. Azathioprine was stopped and the patient was treated with oral prednisolone (30 mg once daily followed by a steroid taper) and colchicine (500 mcg twice daily) [9]. Within 4 weeks, the rash had completely resolved (Fig. 1c, d) and his inflammatory markers normalised. As the onset of symptoms were temporally related to initiation of AZA, a diagnosis of drug-induced Sweet syndrome. His steroid dose was tapered gradually to zero and treatment with colchicine was continued. As of August 2017, his vasculitis remains quiescent and there has been no recurrence of Sweet syndrome, however his ANCA titres remain elevated with anti-MPO levels 170 RU/mL.

## Discussion

**Fig. 2:**
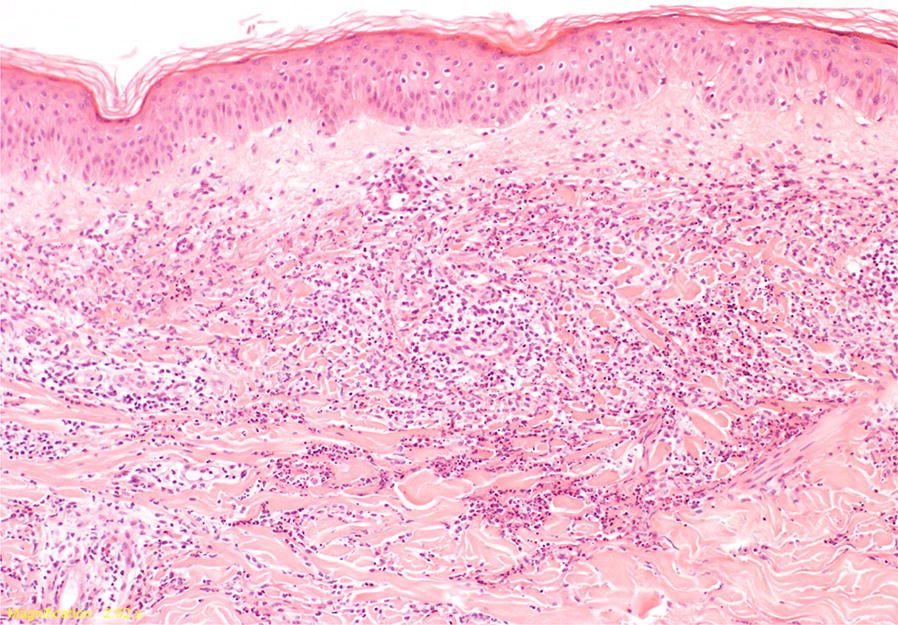
Histopathology of skin biopsy: skin with normal epidermis. There is a diffuse neutrophilic dermal infiltrate with papillary dermal oedema and no evidence of acute vasculitis

We highlight the positive association of MPA with the presence of Sweet’s syndrome in this case report, which is characterised by an intense neutrophilic infiltrate of the dermis. The combination of neutrophilic dermatoses and ANCA-V has only been described in a few case reports. A recent retrospective multicentre study, by the French Internal Medicine Society (SNFMI) and the French Vasculitis Study Group (FVSG), found that Sweets syndrome occurred in three of four patients with MPA suggesting a strong association [5]. It is noteworthy from this review that Sweets syndrome pre-dated the onset of ANCA disease in some patients, and either occurred contemporaneously or followed the onset of ANCA disease in others, suggesting possible external triggers. Our patient with MPA developed classic features of Sweets syndrome within 2 weeks of exposure to AZA therapy. The close temporal association between the exposure to AZA and the development of the skin manifestations, the subsequent resolution after withdrawal of AZA, and treatment with steroids and colchicine suggests a likely causal relationship. Given the strength of this association, we speculated that the occurrence of Sweets syndrome was primarily a result of AZA rather than the underlying ANCA vasculitis. AZA is known to cause many adverse reactions including a well described entity known as azathioprine hypersensitivity syndrome (AHS), which commonly presents as an acute febrile neutrophilic dermatosis. It is difficult to distinguish between AHS and AZA induced Sweet syndrome as they have a very similar presentation [4]. While our case shares many features with AHS, the morphology together with the distribution of the lesions predominantly on the head, neck, and trunk and the biopsy findings of dense neutrophilic infiltrate with marked papillary oedema (Fig. 2) lead us to interpret it as AZA induced Sweets syndrome rather than Sweet’s like or azathioprine hypersensitivity syndrome. Given the similarities between the two entities and the fatal outcomes of AHS, re-challenge of the drug should be avoided. From literature review, we identified two previous cases of AZA-induced Sweet syndrome where ANCA vasculitis was the underlying condition [6, 7]. In each case, patients were positive for MPO-ANCA with evidence of renal involvement and treated with cyclophosphamide. Moreover, the skin rash appeared with commencement of AZA therapy, and in both cases complete resolution occurred on stopping the drug along with treatment with corticosteroids. Despite these strong temporal associations, there are some case reports where Sweets syndrome was diagnosed at the first presentation of ANCA vasculitis and without exposure to medication suggesting a strong linkage with ANCA disease [5].

Sweet syndrome is classified into three types: (1) classical Sweet syndrome, (2) malignancy associated Sweet syndrome and (3) drug induced Sweet syndrome [10]. The criteria for drug-induced Sweet syndrome include the following: (1) abrupt onset of painful erythematous plaques, (2) histopathologic evidence of dense neutrophilic infiltrate without evidence of leukocytoclastic vasculitis, (3) temperature higher than 38 °C, (4) temporal relationship between drug exposure and clinical presentation, and (5) temporal resolution of lesions after drug withdrawal [11]. In our case, the skin rash appeared 2 weeks after commencement of AZA therapy, and resolved after cessation of therapy and a short course of steroids. This temporality of events suggests that AZA was the culprit rather than the ANCA vasculitis, which preceded the skin syndrome by at least 6 months.

## Conclusion

We report a case of Sweets syndrome following exposure to AZA in a patient with pre-existing ANCA vasculitis. Although rare, it is prudent to consider AZA-induced Sweet syndrome in patients presenting with fever and rash within weeks of initiating AZA therapy after excluding infection, malignancy and acute flare of vasculitis. Moreover, the role of ANCA antibodies cannot be completely ignored, and prudence would suggest that patients presenting with Sweets syndrome should be comprehensively investigated for underlying ANCA vasculitis.

## Abbreviations

ANCA: anti-neutrophil cytoplasmic antibodies
ANCA-V: anti-neutrophil cytoplasmic antibody-associated vasculitis
AZA: azathioprine
TBMD: thin basement membrane disease
